# Balancing risk and benefit of SARS-CoV-2 vaccines in children

**DOI:** 10.1016/j.lanepe.2022.100412

**Published:** 2022-05-29

**Authors:** Michael Levin, Elizabeth Whittaker

**Affiliations:** Department of Infectious Diseases, Imperial College London and Department of Paediatircs, Imperial College NHS Trust, London, UK

SARS-CoV-2 vaccines have been rapidly adopted and rolled out to adult populations world -wide. However, vaccination of children has been introduced less rapidly and adopted inconsistently by different countries .This is because relatively few children develop severe COVID, in comparison to the frequency of severe and fatal disease in adults of increasing age.^1^ These risk differences makes balancing potential benefits and risks of childhood vaccination difficult.

One of the major factors supporting roll out of SARS-CoV-2 vaccines to children is their potential to prevent childhood post infectious inflammatory conditions, including the multisystem inflammatory syndrome in children (MIS-C).^2^ MIS-C was first described as the first wave of the COVID19 pandemic swept through Europe and the USA^2^^,^^3^ and is now established as an important severe illness affecting children globally. MIS-C occurs 2-6 weeks after SARS-CoV-2 infection and presents with persistent fever, raised inflammatory markers, often with rash and conjunctival injection. Severe cases progress to shock and multi organ failure. The mechanisms underlying MIS-C are poorly understood, but lack of detectable virus in most cases, and timing after infection suggest an abnormal acquired immune response.

MIS-C is responsible for a significant proportion of admissions to paediatric intensive care units during sucesive COVID-19 waves.^4^ Thus evidence that vaccination against SARS-CoV-2 also prevents MIS-C would be an important factor supporting adoption of SARS-CoV-2 vaccines in children. Conversely evidence of serious complications of the vaccines might shift the balance away from vaccination. Early safety reports from the USA, and France, both countries that have adopted SARS-CoV-2 vaccines for children, have been reassuring, and have shown that vaccines were effective in preventing both severe COVID-19, and MIS- C.^5^^,^^6^ However, recent reports of MIS-C occurring after SARS-CoV-2 vaccination^7^^,^^8^ together with the established link between SARS-CoV-2 vaccines and myocarditis in children,^9^ has increased concern as to the risks of vaccination. What is needed to inform decisions on whether or not to vaccinate children is data from large scale studies on the relative frequency of MIS-C after natural infection and vaccination.

In this issue of *The Lancet Regional Health-Europe*, Ouldali and colleagues^10^ report results of a French national post-authorisation and pharmacovigilance study comparing rates of MIS-C and myocarditis following SARS-CoV-2 mRNA vaccines, administered to 12-17 year old children, as compared with rates of MIS-C following natural infection

Reporting of adverse drug reactions is compulsory in France. Authors used a network of 31 pharmacovigilance centres covering all regions of France to identify cases of MIS-C between June 15th 2021 and January 1 2022, as childhood SARS-CoV-2 vaccination was introduced nationally. Reported cases were rigorously reviewed by a multidisciplinary panel to establish the relationship to previous vaccine administration. Efforts were made to distinguish between vaccine and infection triggered MIS-C by measurement of both nucleocapsid and spike protein antibodies, results of viral PCR and detailed history of exposure. Rates of MIS-C following vaccination were compared with rates of MIS-C following natural infection, utilising national data from mandatory reporting of MIS-C cases and SARS-CoV-2 antibody seroprevalence studies coordinated by Public health France.

The study identified 102 cases of myocarditis or pericarditis and 12 cases of MIS-C following over 8 million doses of SARS-CoV-2 mRNA vaccine administered to 12-17-year-old children. Four of the reported MIS-C cases had evidence of previous SARS-CoV-2 infection, but the remaining 8 had no evidence of infection, and were thus attributed to vaccination. The rates of MIS-C and myocarditis were calculated as 1.5 and 12.6 per million doses of vaccine respectively. In comparison, the rate of MIS-C after natural infection was 113.3 per million SARS-CoV-2 infections. The authors also compared the clinical and laboratory features of post vaccine and infection associated MIS-C and found that post vaccine MIS-C had lower CRP, less requirement for intensive care, but a higher rate of cytolytic hepatitis.

This rigorous study confirms a link between SARS-CoV-2 mRNA vaccines and occurrence of MIS-C and myocarditis in children aged 12-17. However, rates of both of these complications are low compared with the rate of MIS-C after natural infection. As the mRNA vaccines appear to be effective in preventing MIS-C as well as severe COVID ^5^^,^^6^ this study provides reassuring data that will be of help to policy makers, parents and paediatricians. Despite vaccines being associated with rare inflammatory complications, the risk benefit ratio for SARS-CoV-2 vaccination continues to favour vaccination, at least in the 12-17 year old age group included in this study. The finding of a link between vaccination and MIS-C may also provide a clue to the biological mechanisms underlying MIS-C, as it suggests a prominent role for the spike protein, as the sole viral protein expressed by the mRNA vaccines.

Ouldali and colleagues should be congratulated for conducting such a large scale, rigorous and important study which has provided clear evidence on which to base policy.

However SARS-CoV-2 is a moving target, and the balance between vaccine induced and infection induced MIS-C and myocarditis, and the efficacy of vaccines, may be different for Omicron and other new variant waves that seem likely to arise. Furthermore, the duration of protection against both MIS-C and severe COVID-19 following vaccination is unknown. Continued surveillance and monitoring of both SARS-CoV-2 infection rates and severity, and complications of vaccines will be needed to guide decisions on how to use the available vaccines in children.

## Contributors

Both authors contributed equally.

## Declaration of interests

The Authors have no competing or conflicts of interest.
